# Analyzing the Disaster Preparedness Capability of Local Government Using AHP: Zhengzhou 7.20 Rainstorm Disaster

**DOI:** 10.3390/ijerph20020952

**Published:** 2023-01-04

**Authors:** Linpei Zhai, Jae Eun Lee

**Affiliations:** Department of Public Administration & National Crisisonomy Institute, Chungbuk National University, Cheongju 28644, Republic of Korea

**Keywords:** disaster preparedness capability, heavy rainstorm, local government, AHP, evaluation index system

## Abstract

This study aimed to identify factors influencing disaster preparedness capability, measure and compare the relative importance of evaluation indicators of preparedness capability in a rainstorm disaster, and analyze the impact of these factors on disaster preparedness so as to improve disaster preparedness capability. The evaluation model was proposed by constructing the target level (the first level) as an indicator system; this was divided into four indicators (the second level): planning, organization, equipment, and education and exercise, and 14 tertiary evaluation indicators (the third level). The validity of the evaluation index system was demonstrated, and the weight of each level was calculated using the Analytic Hierarchical Process and expert survey methods, taking the example of the Zhengzhou “7.20” rainstorm to conduct an empirical analysis of the proposed model. The weak points of disaster preparedness capability were identified. The empirical analysis revealed that organization scored the highest, followed by planning, equipment, and education and exercise, indicating the lack of disaster management equipment and resources, disaster management training, and exercise and public emergency safety education. These results will help in future decision-making, as they provide a clear understanding of what needs to be done to improve disaster preparedness capability.

## 1. Introduction

Nowadays, various types of emergencies, such as natural disasters, public health crises, and social safety disasters occur frequently, and the importance of emergency response and disaster management has increased significantly in countries around the world. A key task of emergency management is ensuring that the public is adequately prepared for an impending disaster to minimize the loss of life and property [1]. In developing countries, environmental degradation, rapid urbanization, disaster scale, population density, preparedness, and mitigation measures are the main factors affecting disaster-related damage and mortality [2]. China’s new urbanization process has continued to accelerate, with cities becoming larger and attracting an increasing number of people in recent years. With the progress of urbanization, Chinese cities are constantly exposed to a variety of unexpected disasters, including geological disasters (e.g., the Wenchuan earthquake in 2008), meteorological disasters (e.g., Super Typhoon Moranti in 2016, extensive haze and heavy rainfall-related flooding in many places in recent years), fire disasters, traffic disasters, accidents (collapse of self-built houses in Changsha in 2022), and infectious diseases (e.g., SARS from winter 2002 to spring 2003 and COVID-19 in 2020). Many cities suffered catastrophic consequences, such as human casualties, property damage, urban function failure, and social order imbalance as a result of such disasters.

Between 17 and 23 July 2021, China’s Henan Province was struck by an extraordinarily heavy rainstorm that caused severe flooding. The event was named the “7·20” Zhengzhou rainstorm. The “Investigation Report of the ‘7·20’ Extraordinary Rainstorm Disaster in Zhengzhou, Henan Province” labelled the “7.20” rainstorm as a natural disaster triggering severe floods in cities and rivers and causing multiple other disasters, such as landslides, building collapses, and subway accidents, resulting in major casualties and property damage and changing the lives of millions of people [3]. According to verified sources, 14,786,000 people were affected, with direct economic losses of 120.6 billion RMB as of September 30; in Henan Province, 398 people died or were reported missing due to the disaster. The “7.20” Zhengzhou rainstorm disaster was labelled an overall “natural disaster”, and, specifically, a “man-made disaster”. Zhengzhou’s municipal government and relevant districts, counties, departments, and units had poor understanding, risk awareness, and preparedness, with weak preventive measures in place to tackle such a mega-disaster. Moreover, there was dereliction of duty and malfeasance in the emergency response. Local governments play an important role in disaster management because they know their communities and citizens well. When a disaster occurs, local governments should be the first on the scene but, unfortunately, they remain one of the least studied institutions in disaster management literature [4]. Cities’ ability to cope with unexpected disasters need to be improved, and disaster prevention and mitigation strategies pose an urgent problem facing governments at all levels and sectors of society.

Disaster preparedness has been recognized as a critical element in reducing the impact of disasters worldwide [5]. It has been studied from different perspectives and contexts related to individuals [6,7], households [8,9], and communities [10,11]. For example, by integrating community/individual behavior for disaster preparedness [12], certain populations, households, and individuals were found to have different preparedness needs and vulnerabilities [13].

While the literature on disaster risk, disaster resilience, disaster policy and management are relatively mature, public managers are unaware of how to design effective preparedness programs [14]. Few studies explain disaster management capability or disaster governance capability as a key aspect of central and local governments’ disaster management; moreover, studies examining the role of local governments, especially in terms of disaster preparedness, are scarce. It is noteworthy that the primary responsibility for preparedness planning and response, in most cases, lies with the municipality or city [15]. There are two important areas that have not been fully explored with respect to the role of local governments in disaster and emergency management. First, although current research has focused on local governments in developed countries, research on local governments in developing countries is far from adequate. Second, in recent years, many local government agencies seem to be overwhelmed, rushed, and facing difficulties in responding to disasters and reducing related losses, especially in developing countries. The preparedness of local governments to manage disasters at each stage (before, during, and after) has not yet been tested. 

The purpose of this study is to identify the factors that influence disaster preparedness capability, measure and compare the relative importance of evaluation indicators of preparedness capability in a rainstorm disaster, and analyze the impact of these factors on disaster preparedness to improve disaster preparedness capability. We also analyze relevant literature, refer to previous research results, combine the expert survey and the Analytic Hierarchical Process (AHP) methods, refine and construct a comprehensive disaster preparedness assessment system, develop a disaster preparedness assessment capability model, assign weights to indicators, and finally take the example of the “7·20” rainstorm in Zhengzhou city to conduct an empirical analysis of the proposed model. Through this comprehensive assessment, we aim to determine the weak points of disaster preparedness capability.

## 2. Theoretical Background

### 2.1. Disaster Preparedness

Social scientists, emergency managers, and public policymakers generally study and guide the process of disaster occurrence around four phases: mitigation, preparedness, response, and recovery [16]. This prevention phase of disaster management can be defined as all activities that can be implemented by the population, government, and relief organizations before a disaster occurs, with the aim of reducing its potentially devastating effects [17]. Preparedness is not only a state of readiness, but also a theme throughout most aspects of emergency management. It should be a dynamic and continuous management process, directly affecting the performance of emergency response capabilities, thus determining the development and evolution of the situation [18]. Preparedness comprises measures that enable different units of analysis—individuals, households, organizations, communities, and societies—to respond effectively and recover more quickly when disasters strike [16]. It is the ability of people to (a) anticipate what they have to deal with (dangerous consequences); (b) respond to, adapt to, and recover from disaster-related consequences, especially in areas that are likely to experience repeated disaster events; and (c) learn from these experiences [19] (p. 46).

Natural disaster preparedness is generally considered the preferred mechanism to encourage proactive activities (behavioral, cultural, structural, or institutional) to mitigate the disastrous potential of these events [20]. Preparedness has dual objectives: to reduce vulnerability to a potential threat [21,22,23] and to increase the resilience of the public exposed to a threat [24,25,26]. Activities that are commonly associated with disaster preparedness include developing planning processes to ensure readiness, formulating disaster plans [27], stockpiling resources necessary for an effective response [28], and developing skills [8] and competencies to ensure effective performance of disaster-related tasks [16]. 

Many previous studies have revealed that preparedness factors contribute to differences in disaster preparedness levels [27,29,30], such as personal, family, and social factors, and selective measures of preparedness. Residents’ personal disaster preparedness refers to the actions taken in response to disasters and loss reduction [31]; it is also known as risk perception [32,33]. Physiological activities, such as attitudes and beliefs thus alter people’s hazard avoidance behavior [34], as do the previous experience and knowledge of hazards [35,36], disaster preparedness knowledge [37], and access to information sources [38]. Social factors include social networking [8] and trust in the government [30,38]. These studies considered a range of dimensions and different measures of preparedness.

In summary, disaster preparedness is a series of activities implemented to mitigate possible damage and reduce the adverse effects of a disaster. It is not only a part of the crisis and emergency management activities according to the time division, but also a fundamental action throughout the crisis and emergency management process that is performed before, during, and after the disaster.

### 2.2. Components of Disaster Preparedness Capability

In the case of disasters, it is critical to identify the changing needs of the disaster response environment and to foster the management capability needed to respond to disasters. Capability operational transformation is a critical success factor for disaster management [39]. Cigler [40] defines capability as the financial, technical, policy-related, institutional, leadership, and human resource capabilities that local government agencies must have in order to operate in all phases of daily emergency and disaster situations. The capability required for disaster management is related to the delegation of authority, communication, decision-making, and inter-agency coordination [41]. Common mistakes that local governments make in preventing disasters are often related to rigid institutional beliefs, ignoring external complaints, difficulties dealing with multiple sources of information, and a tendency to minimize danger [42]. Therefore, disaster preparedness often requires coordination between individuals, governments, agencies, and organizations to improve training and exercise plans, enhance and introduce technological innovations, and ensure that individuals, social organizations, and businesses in various fields support this ability.

This paper is based on the preparedness capability elements classified by the Federal Emergency Management Agency (FEMA) National Preparedness Directorate, and the components of disaster preparedness capability are shown in Table 1. It shows the planning process that begins with planning for the various hazards that exist, and then builds and improves readiness systematically. This cycle recognizes the importance of the four main components of any preparation: planning, organization, equipment, and education and exercise. This cycle represents not only readiness at all levels of government jurisdictions, but also readiness actions taken by individuals, businesses, NGOs, and other entities [18,43].

### 2.3. Comprehensive AHP Evaluation Model

**Figure 1:**
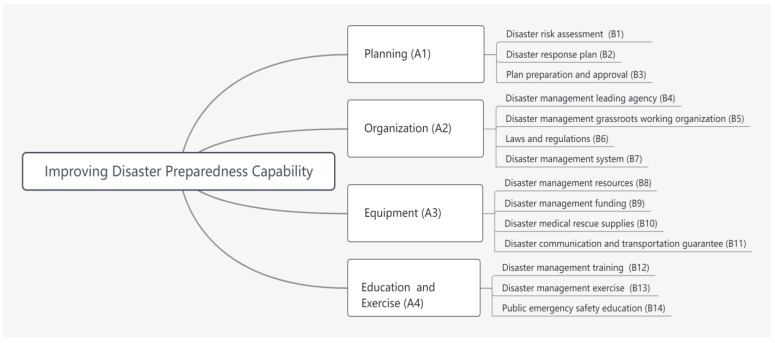
Comprehensive evaluation model of the disaster preparedness capability index system.

Based on an extensive data research and literature review, we developed a three-level AHP evaluation model with disaster preparedness capability as the target with reference to the US FEMA, as shown in Figure 1. In the AHP model, the target level is the disaster preparedness capability. The evaluation indices of disaster preparedness are divided into four second-level indicators: planning (A1), organization (A2), equipment (A3), and education and exercise (A4), and 14 tertiary evaluation indicators: disaster risk assessment (B1), disaster response plan (B2), plan preparation and approval (B3), disaster management leading agency (B4), disaster management grassroots working organization (B5), laws and regulations (B6), disaster management system (B7), disaster management resources (B8), disaster management funding (B9), disaster medical rescue supplies (B10), disaster communication and transportation guarantee (B11), disaster management training (B12), disaster management exercise (B13), and public emergency safety education (B14). 

## 3. Materials and Methods

The Analytic Hierarchical Process, proposed by American operations researcher Saaty in the 1970s, is a comprehensive weighted decision-making method that uses mathematics and psychology to organize and analyze complex decisions, assigning weights in the process of comparing the relative importance of indicators to ensure that a logically consistent solution is reached. It is applicable to decision-making problems involving complex hierarchies and multiple indicators [44]. As a decision system, AHP is valuable for using human cognition to determine the relative importance between a set of alternatives through pairwise comparisons [45]. This approach has been used in various studies aimed at promoting development in different sectors, such as environment and natural resources [46]; disaster management, disaster resilience, and vulnerability indices [47,48,49,50]. Therefore, in this study, AHP was used to not only identify the criteria and influencing factors that best describe disaster preparedness, but also evaluate the importance and priority among indicators, and develop a tool to quantify disaster preparedness capability. 

**Figure 2:**
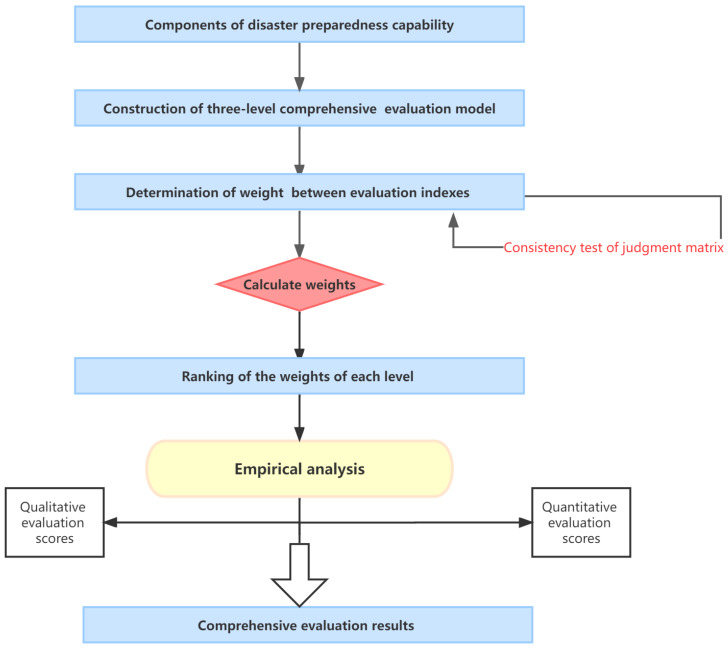
Calculation process chart.

The use of an AHP analysis to determine the evaluation index system and the weights can be divided into the following steps: establishing the hierarchical structure according to the hierarchical relationship, constructing a judgment matrix, calculating the judgment matrix to obtain the relative weights of the evaluation indices, and testing the consistency of judgment to obtain the final weights of indices at each level. On this basis, we take the Zhengzhou “7·20” rainstorm as an example to conduct empirical analysis; carry out qualitative and quantitative empirical analysis of emergency preparedness ability; obtain the scores of various indicators; use EXCEL and SPSS software 26 to input and process score data, respectively; and obtain the final comprehensive evaluation results. The specific steps and process are shown in Figure 2.

To ensure the objectivity of the relevant data obtained and to scientifically determine and rank the importance of the weights of the indicators to ensure the validity of the indicator system, this paper solicited and obtained data of the weights of each indicator by issuing questionnaires to 14 experts and scholars in the field of government disaster management who were recommended by professors and contacted directly. They were viewed as decision-makers in the prioritization process, making evaluations and choices based on their experience, skill, knowledge, and practice [49]. The questionnaire data were collected from 17 to 20 September 2022. All 14 completed questionnaires were collected. The basic information of the survey respondents is shown in Table 2. The questionnaire used the scale method of 1~9 and their reciprocals. The complex problem was broken down, level by level, and the indicators in the hierarchy were compared in terms of their relative importance in determining their overall order of importance.

### 3.1. Determination of Weight Value between Evaluation Indices

In this paper, we used YAAHP software 12.8 to calculate the weights for each level of indicator (i.e., the degree of importance, according to the abovementioned steps). The index weights were calculated according to AHP and the weights of each hierarchical evaluation index system were also calculated; the results are shown in Table 3. Four indicators were evaluated at the second level: (A1), (A2), (A3) and (A4) with weights of 0.294, 0.220, 0.257, and 0.228, respectively. 

In the consistency test, the consistency ratio (CR) is generally within 0.1, suggesting that the calculation results are consistent and that the consistency of the judgment matrix is acceptable [51] (p. 287). However, in some cases, 0.2, but never more, is tolerable [52] (p. 34). According to the software’s calculation results, the consistency index (CI) and the average random consistency index (RI) are derived, and the consistency ratio (CR) is finally calculated as follows: CR = CI/RI. The analysis results of the AHP model in the disaster preparedness index system revealed that the CR of disaster preparedness = 0.077 < 0.1, which meets the consistency requirement. Meanwhile, the CRs of planning (A1), organization (A2), equipment (A3), and education and exercise (A4) were 0.055, 0.079, 0.038, and 0.076, respectively, suggesting that the constructed judgment matrix had a high degree of consistency.

### 3.2. Composite Weight Ranking of Judgment Matrix

**Figure 3:**
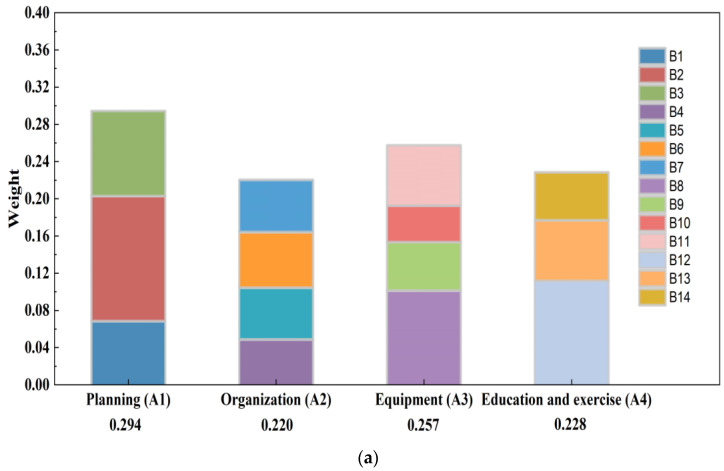

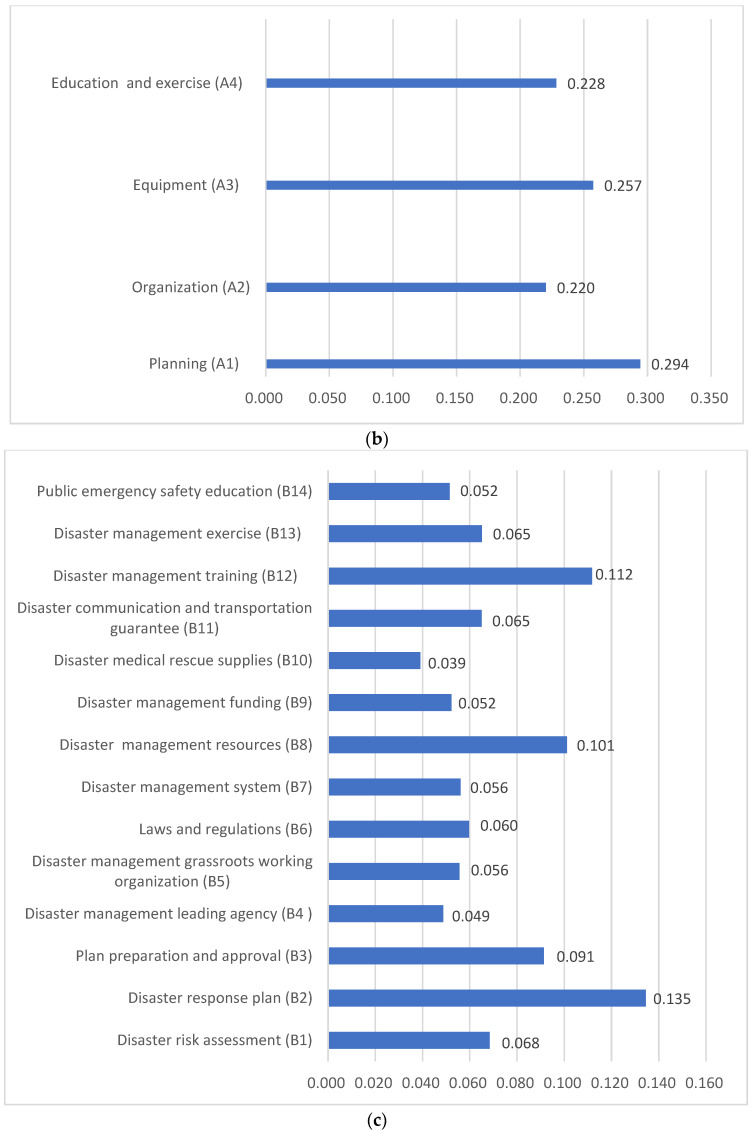
(**a**) Comparisons between different indicators at each level. (**b**) The weight ranking of the second level (A1~A4) to the target level. (**c**) The weight ranking of the third level (B1~B14) to the target level.

Figure 3a shows the weights of the different indicators at each level, including comparisons between levels. Combined with the hierarchical relationships of the indicators in the constructed model, different weight distributions of the different indicators affecting the disaster preparedness capability can be seen. The weight ranking of the criterion layer (second-level) and scheme layer (third-level) to the target layer is shown in Figure 3b,c, respectively. In Figure 3b, Planning (A1) had the largest weight in the overall disaster preparedness capability, followed by equipment (A3), education and exercise (A4), and finally, organization (A2), which accounted for the smallest weight, while training and education and exercise had the least influence on the overall disaster preparedness capability. In Figure 3c, compared with other indicators, the disaster response plan (B2) was the most important for assessing overall disaster preparedness capability, indicating that the prevention work plan before an incident was critical to overall disaster preparedness. When hazardous accidents and disasters occur, we must target our disaster preparedness efforts toward improving emergency rescue and disposal capabilities and minimize losses. Additionally, disaster management training (B12), disaster management resources (B8), and plan preparation and approval (B3) had a greater impact on disaster preparedness. The three with the least impact were public emergency safety education (B14), disaster management leading agency (B4), and disaster medical rescue supplies (B10).

### 3.3. Relative Weight Ranking of Judgment Matrix

**Figure 4:**
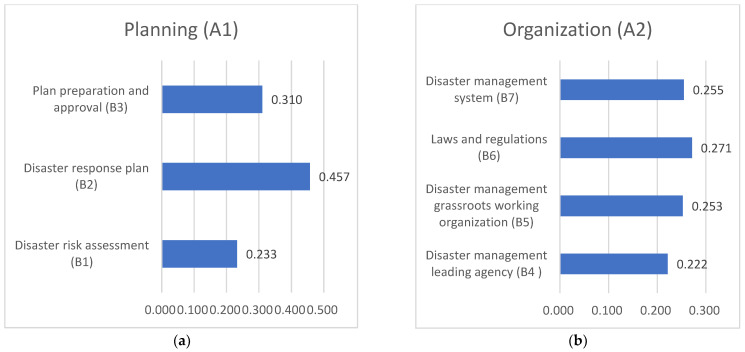

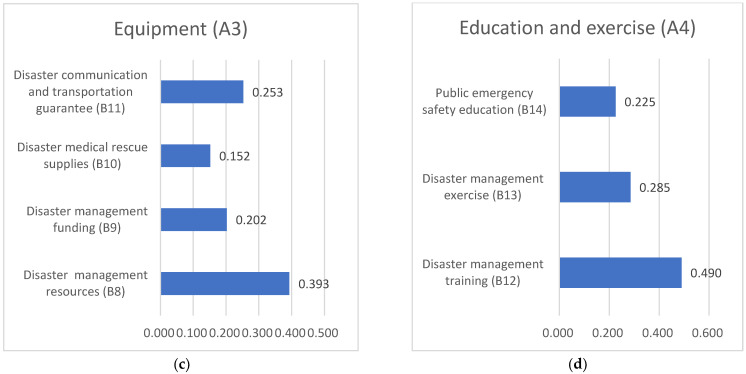
(**a**) The weight ranking of the third level (B1~B3) to the second level A1. (**b**) The weight ranking of the third level (B4~B7) to the second level A2. (**c**) The weight ranking of the third level (B8~B11) to the second level A3. (**d**) The weight ranking of the third level (B12~B14) to the second level A4.

According to the judgment matrix, the relative weight ranking of the third level to the corresponding second level can be obtained separately (Figure 4). For planning (A1), disaster response plan (B2) had the largest weight (45.7%), followed by plan preparation and approval (B3) (31%), and disaster risk assessment (B1) (23.3%). Thus, the disaster response plan greatly impacted planning (see Figure 4a). For organization (A2), laws and regulations (weighted at 27.1%) and disaster management systems (weighted at 28.4%) were more important. Additionally, the proportions of disaster management grassroots working organizations and disaster management leading agencies to the organization were 25.3% and 22.2%, respectively (Figure 4b). For equipment (A3), the weight ratio of disaster resources was as high as 39.3%, followed by disaster communication and a transportation guarantee with a weight ratio of 25.3% (both of which directly affected equipment capabilities), disaster funding (weighted at 20.2%), and finally, disaster medical rescue supplies (weighted at 15.2% (see Figure 4c). For education and exercise (A4), disaster management training directly affected education and exercise capability, with a weight ratio of 49%, followed by a disaster management exercise at 28.5% and public emergency safety education at 22.5% (see Figure 4d).

## 4. Results

### 4.1. Empirical Analysis

The abovementioned evaluation method was used to assess the disaster preparedness of Zhengzhou city for this “7·20” rainstorm by combining the disaster preparedness evaluation index system and the actual disaster preparedness in response to the “7·20” rainstorm. Twenty experts engaged in government disaster management or staff of relevant government departments were selected as the subjects, and 19 valid questionnaires were finally collected, with a valid return rate of 95%. The questionnaire sought to determine contents of the three-level indicators, which were divided into quantitative and qualitative indicators according to the form of the basic data obtained from the statistical indicators. Therefore, excluding the first part of basic personal information, the questionnaire subjects were divided into two main blocks based on quantitative and qualitative content. Quantitative indicators can be judged by specific numerical values, such as the number of personnel, ambulance supplies, and shelters. Qualitative indicators were values of indicators that cannot be expressed by specific numbers, and questionnaire participants often provide descriptive data based on intuition or experience. For the convenience of calculation, a five-point Likert scale was used to convert the graded values into statistically significant indicators. We used Excel and SPSS software 26 to organize, enter, and calculate the obtained data, and descriptive statistical analysis was performed, resulting in scores of qualitative and quantitative indicators, respectively. Next, the qualitative evaluation and quantitative evaluation scores were added together and divided by 2 to obtain the comprehensive scores. A reliability test was conducted based on the results and the Cronbach’s alpha was 0.993, indicating the scientific credibility of the findings.

**Figure 5:**
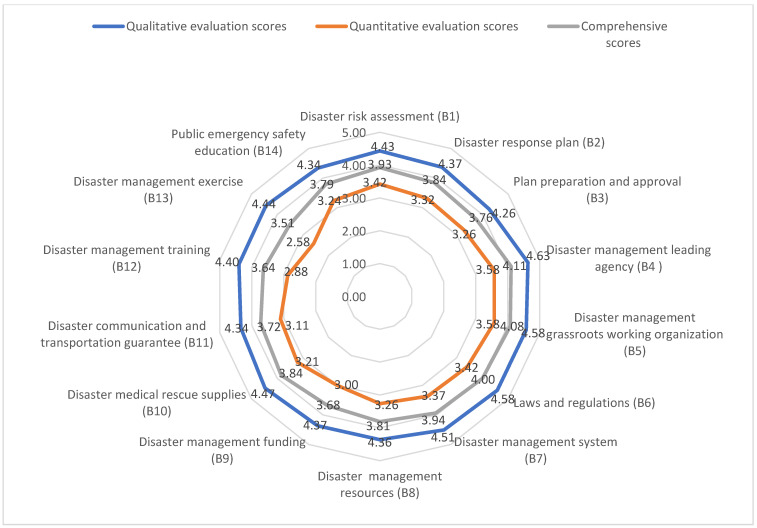
Radar chat of qualitative evaluation scores, quantitative evaluation scores, and comprehensive scores of B1~B14.

Figure 5 shows the qualitative evaluation scores, quantitative evaluation scores, and comprehensive scores of B1~B14. After the collation and calculation, in terms of qualitative evaluation scores, the disaster management leading agency (B4) had the highest score, followed by the disaster management grassroots working organization (B5), and laws and regulations (B6); plan preparation and approval (B3) had the lowest score. The qualitative assessment of disaster preparedness revealed more recognized scores of disaster response organization, while the disaster management leading agency and grassroots working organization were more recognized; the related ability of disaster plan preparation and public emergency safety education was somewhat inadequate. The quantitative evaluation scores for B1 to B14 were 3.42, 3.32, 3.26, 3.58, 3.58, 3.42, 3.37, 3.26, 3.00, 3.21, 3.11, 2.88, 2.58 and 3.24, respectively, with the disaster management leading agency (B4) and disaster management grassroots working organization (B5) having the highest scores. This is consistent with the qualitative assessment score, followed by disaster risk assessment (B1) and laws and regulations (B6); disaster management exercise (B13) had the lowest score. Thus, in the quantitative assessment of disaster preparedness, the performance of disaster response organization and disaster response regime was more recognized, and the related ability of disaster management exercise and disaster management training was somewhat lacking.

Comprehensive scores of the individual index reveal that disaster management leading agency (B4) had the highest score in both qualitative and quantitative assessments, followed by the disaster management grassroots working organization (B5), and laws and regulations (B6). The last three rankings were disaster management exercise (B13), disaster management training (B12), and disaster management funding (B9), indicating that Zhengzhou city needs to pay more attention to disaster training, exercise, and disaster management funding in the future.

### 4.2. Comprehensive Evaluation Results

Overall, the combined assessment scores for the second-level indicators were 3.84, 4.03, 3.77, and 3.65 (out of 5) (see Table 4). The highest score for organization (A2) indicated that Zhengzhou had a more complete disaster management organization and system, followed by planning (A1), and finally, equipment (A3), and education and exercise (A4), indicating a major lack of daily disaster management training, exercise, and public emergency safety education areas in Zhengzhou. Specifically, the qualitative assessment scores of the secondary indicators were higher than the quantitative assessment scores, indicating that the Zhengzhou government had a clear understanding of the content and objectives of the work needed to improve disaster preparedness but was not sufficiently concerned about the implementation of tasks. The relevant authorities should be urged to strengthen the supervision and management of the implementation of the entire disaster preparedness process.

In conclusion, there were still some shortcomings in Zhengzhou City’s preparation and response to the extraordinarily heavy “7·20” rainstorm. First, there was insufficient awareness of major hazard and threat information and poor awareness of disaster risk; the person in charge had a subjective sense of judgment, lacked sensitivity and alertness to major hazard signals, and ignored the forecast information made by the meteorological department. Second, there was an obvious disconnect between emergency operations and forecast information dissemination and no quick or timely alert announcement information to the society. Third, the formulation, evaluation, and revision of the plan were not refined, and the practice was not strengthened. In many disaster-prone areas, local and national governments and NGOs have worked to provide disaster education programs and emergency training to raise awareness and promote self-reliance and family preparedness [53]. Thus, the process of responding to this extraordinarily heavy rainstorm revealed that the dissemination of disaster warning information was not timely or adequate and that safety awareness and disaster prevention and avoidance capabilities were weak. The disaster education knowledge of leaders at all levels, disaster management capability training, and safety knowledge education for the public should all be improved.

## 5. Discussion

Based on the scores of the comprehensive evaluation indicators and the level of disaster risk in Zhengzhou, the following suggestions are provided for disaster preparedness in Zhengzhou: In terms of planning (A1), an important aspect of disaster planning is to convey to the public the nature of the risk and make appropriate adaptation strategies so that people have a clear perception of risk and know what to do and what not to do before and after a disaster [54]. We assume that all disasters are local and that the primary responsibility for managing disasters and emergencies, including informing and alerting the public, belongs to local governments [55]. An effective system requires that early warning information [1] and risk reduction be mainstreamed into the policy process and that government agencies have the capability to design and implement effective policies. Effective early warning system policy processes also require the involvement of local communities to ensure that the at-risk public is adequately informed and alerted [56]. Communities and residents are responsible for taking their own measures to prepare for disasters, and the final decision on disaster preparedness measures rests with individuals [57]. The public takes disasters more seriously when they have a large amount of information and credible disaster warnings [58].Thus, information collection on major hazards and threats should be increased, the sensitivity of major hazard signals should be maintained, the upgrading of the monitoring and warning information platform and release system should be accelerated, and multi-source information should be combined and processed quickly and efficiently to ensure the timely release of warning information to the community at the first instance of an accident. Simultaneously, to further improve the system, we should pay attention to updating and enhancing the disaster management plan, which should be filed with relevant superior departments or agencies, in order to enhance the integrity, coordination, and effectiveness of the system.

In terms of the organization (A2), we should continue to improve the disaster management system and institutional set-up to ensure disaster preparedness in an effective and organized manner. We should also enhance the efficiency of disaster management departments, while improving the disaster command and coordination mechanism to ensure coordination and linkage among functional departments and different administrative regions in an orderly and efficient manner. Additionally, the construction of a comprehensive disaster rescue system, disaster management leadership, and cooperation between the leading agencies of disaster management and local working agencies should be enhanced.

In terms of the equipment (A3), resources are stable assets that can be used to deal with a variety of situations, including those related to health, income, and social support. Having disaster resources is critical to proactively respond to disasters and crises [59]. It is necessary to focus on strengthening the provision and maintenance of disaster relief equipment and materials. Furthermore, we should increase the construction of emergency shelters, open up qualified gymnasiums, parks, and other places that can serve as emergency shelters, equip them with supporting facilities, strengthen emergency material and fund reserves, and regularly update the facilities and equipment required for living.

In terms of education and exercise (A4), education can trigger a learning process that improves disaster preparedness. In the future, managers will need to be educated on disaster preparedness planning and should work with local agencies to provide disaster training for other managers, teachers, and staff to ensure that appropriate actions are taken to minimize loss of life and property [60]. Disaster education increases coping potential, thereby reducing the adverse effects of anxiety on disaster preparedness [61]. Regular training activities for emergency management-related practitioners as well as general personnel, educational activities, and various forms of emergency plan exercises, will help improve the proportion of practitioners who meet the qualification requirements and the professionalism of rescue teams. Their focus should be on making citizens aware of their own important role and disaster preparedness actions while responding to a disaster or crisis. Due to the limited resources and capabilities of the government, it is impossible to rescue each victim in time, which makes the mutual and self-rescue of citizens very important in the early stage of disasters. Therefore, multi-form and multi-content public safety culture improvement activities should be conducted to enhance their emergency preparedness capability. Additionally in this process, the role of active opinion leaders in disaster preparedness is valued. Opinion leaders can actively organize training on disaster prevention and mitigation knowledge, strengthen public disaster risk awareness and guide the public to properly respond to natural disasters [62].

## 6. Limitations

The existing literature does not provide an official definition of disaster preparedness capability, and an official disaster preparedness assessment system that is applicable to various countries and governments at all levels has not yet been developed. The influencing indicators of capability are relatively complex. Therefore, the division of disaster preparedness tasks and capability described in this paper may be lacking in certain forms, as the construction of indicators is incomplete and the influencing factors are interconnected and mutually restrictive, as has been repeatedly demonstrated in combination with practical applications. 

## 7. Conclusions

In this study, the evaluation model of disaster preparedness capability was proposed, the scores and grades of the indicators were summarized and classified to comprehensively analyze and evaluate the disaster preparedness capability of Zhengzhou city, then some suggestions were put forward. The results revealed that Zhengzhou city residents had a clear understanding of the content and objectives of the work needed to improve disaster preparedness and had a relatively complete disaster management organization and system. However, there was an overall lack of attention to the planning and implementation of tasks, which can further encourage poor management activities [63]. The research results can help find weak links in disaster preparedness in the future and improve the disaster preparedness capability of various disaster response subjects. Disaster drills and training involve many agencies and resources. There is a need to strengthen partnerships with civil society organizations, community volunteers, and local chambers of commerce and industry to build a network of organizational partners in the public, private, and non-profit sectors in disaster management [64]. The resources of these organizations and groups can improve local government weaknesses [65]. Simultaneously, effective cognitive and psychomotor skills should be developed through organized and planned training to deal effectively with disaster situations [66], and relevant departments need to be encouraged to strengthen the supervision and management of the implementation of the whole process of disaster preparedness. Regarding the disaster preparedness assessment framework proposed and constructed in this paper, with the continuous strengthening of the overall level of disaster response work, there may be some changes in the indicators in different countries and scenarios, and research will be conducted in consideration of the actual situation. Therefore, a multi-country comparative analysis will be carried out in the future and will be further improved in follow-up research.

## Figures and Tables

**Table 1:** Components of disaster preparedness capability.

Planning	Disaster management planning at the government level is a necessary and complex process. Governments must know what needs to be done, how they will do it, what equipment will be used, and how they can get other agencies or people to help them. In the event of a disaster, each level of government is required to perform a range of tasks and functions before, during, and after the event. The most comprehensive approach to disaster management planning is the development of a national Emergency Operations Plan (EOP that includes disaster risk assessment (B1), disaster response plan (B2), nd plan preparation and approval (B3). Planning may also include: demand analysis, hazards risk analysis, plan evaluation, revision and improvement, the disaster planning system, hazard identification, and comprehensive evaluation of disaster risks.
Organization	When the government responds to a disaster, it is critical to ensure that all individuals and agencies involved in the emergency management system can perform their responsibilities and have appropriate statutory disaster management organization: disaster management leading agency (B4) and disaster management grassroots working organization (B5) under laws and regulations(B6). EOPs define the actions of specific authorities, and statutory authorities give them the authority to take those actions. Agreements between neighboring communities and even countries, as well as between jurisdictions, contribute to the need for a legal and disaster response system (B7) framework in the same country before a disaster occurs. Examples of organization include: policy guidance, disaster management system, disaster management leading agency, disaster management organization, and expert groups.
Equipment	Developing tools, technologies, and other equipment to assist in disaster response and recovery helps the responding agencies significantly reduce the number of casualties and properties damaged and destroyed as a result of disasters. Disaster rescue equipment also adds to the effectiveness of responding agencies by protecting the lives of responders. This equipment is primarily driven by available disaster management resources (B8), disaster management funding (B9), disaster medical rescue supplies (B10), and disaster communication and transportation guarantee (B11).
Education and exercise	Disaster management training (B12), disaster management exercise (B13), and public emergency safety education(B14) constitute the fourth component of government disaster preparedness capability. Considering disaster management, response officials who are not adequately trained in the details of specialized responses are at serious risk. Untrained or inadequately trained responders increase the likelihood of secondary emergencies or disasters, further contributing to the shortage of response resources. Examples of education and exercise include: training of general personnel, training of disaster response team, qualification certification, public emergency safety education, evaluation of educational activities, disaster exercise, exercise planning, and exercise evaluation.

Source: Adapted from Coppola [43] (pp. 276–296).

**Table 2:** Descriptive statistics of survey participants.

Characteristics		Frequency	Characteristics		Frequency
Gender	Male	8	Education	Master’s degree	4
	Female	6		Doctoral degree	10
Age	30–39	6	Number of years of research or work	Less than 5 years	2
	40–49	3		5–10 years	4
	>50	5		More than 10 years	8

**Table 3:** Weight of each index of the disaster preparedness capability evaluation index system.

Second-Level(A)	Weight	Priority	Third-Level(B)	Relative Importance	Priority	Composite Weight	Priority
Planning (A1)	0.294	1	Disaster risk assessment (B1)	0.233	3	0.068	5
			Disaster response plan (B2)	0.457	1	0.135	1
			Plan preparation and approval (B3)	0.310	2	0.091	4
Organization (A2)	0.220	4	Disaster management leading agency (B4)	0.222	4	0.049	13
			Disaster management grassroots working organization (B5)	0.253	3	0.056	10
			Laws and regulations (B6)	0.271	1	0.060	8
			Disaster management system (B7)	0.255	2	0.056	9
Equipment (A3)	0.257	2	Disaster management resources (B8)	0.393	1	0.101	3
			Disaster management funding (B9)	0.202	3	0.052	11
			Disaster medical rescue supplies (B10)	0.152	4	0.039	14
			Disaster communication and transportation guarantee (B11)	0.253	2	0.065	7
Education and exercise (A4)	0.228	3	Disaster management training (B12)	0.490	1	0.112	2
			Disaster management exercise (B13)	0.285	2	0.065	6
			Public emergency safety education (B14)	0.225	3	0.052	12

**Table 4:** Comprehensive scores of second- and third-level indicators.

Second-Level(A)	Third-Level(B)	Qualitative Evaluation Scores	Quantitative Evaluation Scores	Comprehensive Scores	
Planning (A1)	Disaster risk assessment (B1)	4.43	3.42	3.93	3.84
	Disaster response plan (B2)	4.37	3.32	3.84	
	Plan preparation and approval (B3)	4.26	3.26	3.76	
Organization (A2)	Disaster management leading agency (B4)	4.63	3.58	4.11	4.03
	Disaster management grassroots working organization (B5)	4.58	3.58	4.08	
	Laws and regulations (B6)	4.58	3.42	4.00	
	Disaster management system (B7)	4.51	3.37	3.94	
Equipment (A3)	Disaster management resources (B8)	4.36	3.26	3.81	3.77
	Disaster management funding (B9)	4.37	3.00	3.68	
	Disaster medical rescue supplies (B10)	4.47	3.21	3.84	
	Disaster communication and transportation guarantee (B11)	4.34	3.11	3.72	
Education and exercise (A4)	Disaster management training (B12)	4.40	2.88	3.64	3.65
	Disaster management exercise (B13)	4.44	2.58	3.51	
	Public emergency safety education (B14)	4.34	3.24	3.79	

## Data Availability

A copy of the questionnaire and datasets for this study are available from the corresponding author upon well-founded request.
